# Editorial for the Special Issue on Nanostructured Surfaces and Devices for Biomedical Applications

**DOI:** 10.3390/mi13122094

**Published:** 2022-11-28

**Authors:** Valentina Mussi, Annalisa Convertino, Antonella Lisi

**Affiliations:** 1 IMM CNR, Institute of Microelectronics and Microsystems, National Research Council, 00133 Rome, Italy; 2 IFT CNR, Institute of Translational Pharmacology, National Research Council, 00133 Rome, Italy

The ability to control and modify the surface topography of materials at the nanoscale, which produces features with a comparable size to that of biological entities, so as to effectively probe and influence processes at both the cellular and the molecular level, has facilitated incredible possibilities in the fields of biomedicine, biosensing, and diagnostics. After several years of curiosity-driven and successful exploration efforts, it is time to take stock and try to trace a possible future of nanostructured surfaces and more complex micro and nanoscale devices for translational and personalized medicine. This applicative perspective requires on one side the combined use of diverse disciplines and expertise in order to work at the frontier between physics, biology, chemistry, and engineering, on the other a precise identification of medical needs and targets. The Special Issue explores these frontiers and needs, by collating different contributions devoted to the main scientific aspects involved in this innovation course, and proposing possible strategies to push specific structures and methodologies forward from the bench to patient bedsides.

The first important point concerns the dimensional adaptation of structures and devices for the diagnostic target. This element is at the center of the paper by Trejo-Soto and Hernández-Machado [1], which reports on the possibility of using a front micro-rheology technique based on channel microfluidic devices to analyze the biomechanical properties of single erythrocytes, that play a fundamental role in blood viscosity and can be an important indicator of diseases related to RBCs and blood flow (see also [2,3]). However, this size adaptation can go further down the dimensional scale to reach the nanometric size of subcellular compartments and molecules, as demonstrated in papers by Durastanti et al. [4] and Mussi et al. [5], devoted to the exploitation of disordered nanostructured platforms for the optical sensing and recognition of genomic DNA and even single nucleotides (see also [6,7]). Interestingly, the two contributions rely on an order-to-disorder transition of the nanostructure’s arrangement, which is shown to be especially effective for biosensing, and has a clear advantage of reducing fabrication costs and enhancing production throughput. Moreover, the two papers represent an example of approaches where ‘active properties’ of nanostructured substrates are also involved (surface enhancement Raman scattering effect—SERS—which makes it possible to analyze low-concentration biological samples without the need for fluorescent labelling) and multivariate statistical analysis is used to quickly perform data interpretation and classification.

The contribution by Almaviva et al. [8] is developed on a similar basis, whereby a functionalized SERS chip is employed to detect and identify Bacillus thuringiensis cells, a harmless surrogate of the pathogen Bacillus anthracis responsible for the deadly Anthrax disease, and powerful chemometric methods are applied to classify the data with the overarching purpose of attaining an automated recognition procedure and to lay the groundwork for a practical diagnostic use of the chip (see also [9]).

In fact, a concrete final implementation of the proposed platforms constitutes the distinctive characteristic of this Special Issue project, which aims to outline the route to the commercial realization of real nanoscale biomedical devices. Therefore, just as refs. [4,5] propose a disordered disposition of the nanostructures grown by chemical methods to reduce fabrication complexity, the review by Surdo et al. [10] illustrates nanopatterning technologies based on photonic nano-jets having the potential to become a fully controlled, cost-effective, and easy-to-use tool for a wide avail of nanostructured systems in biomedical and theranostic research (see also [11,12]).

The paper by Carotenuto et al. [13] presents an important example of such therapy-oriented systems, i.e., micro/nanostructured scaffold structures obtained through the careful optimization of the biomaterial micro/nano architecture, which can influence cellular behavior and guide cells towards a specific soft or hard tissue organization, thus representing a crucial mimicking strategy for successful regenerative therapy (see also [14,15]).

In the same field of cell investigation and manipulation, extraordinary results have been obtained by realizing scaffolds and biodegradable structures that can reproduce micro and nanoscale natural tissue organizations, as described by the review by Ul Haq et al. [16] which is dedicated to the potential of cardiac tissue engineering by conductive nanomaterials to repair myocardial infarction injuries (see also [17]).

The integration of the dimensional, morphological, and “active” properties of nanomaterials with dedicated statistical and analytical methods thus seems to be the key feature for the biomedical application of nanostructured structures and devices, and it is possible to predict that the panorama of scientific research will grow over the next few years in light of several smart nanostructured systems, that will go on to compose the continuously evolving map of Nanomedicine.
